# Mechanisms of matrix metalloproteinase-2 (mmp-2) transcriptional repression by progesterone in jar choriocarcinoma cells

**DOI:** 10.1186/1477-7827-7-41

**Published:** 2009-05-09

**Authors:** Shlomit Goldman, David H Lovett, Eliezer Shalev

**Affiliations:** 1 Laboratory for Research in Reproductive Sciences, Department of Obstetrics and Gynecology, HaEmek Medical Centre, Afula, Israel; 2 The Department of Medicine, SFVAMC, University of California San Francisco, San Francisco, CA, USA; 3 Rappaport Faculty of Medicine, Technion-Israel Institute of Technology, Haifa, Israel

## Abstract

**Background:**

Although the MMP-2 promoter lacks a canonical progesterone response element (PRE), the hormone inhibits MMP-2 expression and is part of treatment protocols in gynecological invasive pathologies, including endometriosis and endometrial hyperplasia. This study aimed to explore the mechanism by which progesterone inhibits MMP-2 expression.

**Methods:**

The effect of progesterone on MMP-2 expression in the JAR human choriocarcinoma cell line was analyzed by gelatin zymography. MMP-2 transcript expression was studied using Northern blot and semi-quantitative RT-PCR. Rat promoter deletion analysis, electrophoretic mobility shift and chromatin immuno-precipitation assays were performed in order to locate the DNA binding site and the transcription factors involved in MMP-2 regulation.

**Results:**

Progesterone significantly decreased secretion of pro-MMP-2 and MMP-2 transcript expression level in a dose-dependent manner. Progesterone (1 microM) significantly decreased both human and rat MMP-2 promoter activity (80.1% +/- 0.3 and 81.3% +/- 0.23, respectively). Progesterone acts through the SP1 family transcription factors-binding site, located between -1433 and -1342 bp region from the transcriptional start site of the rat MMP-2 promoter, which are present in the orthologous human MMP-2 promoter. Progesterone receptor (PR), SP2, SP3 and SP4 proteins are constitutively bound to this consensus sequence.

**Conclusion:**

Progesterone reducesPR and SP4 binding to the MMP-2 promoter, thereby suppressing transcription. Progesterone also promotes SP4 degradation. These novel mechanisms of MMP-2 regulation by progesterone provide the biological rationale for the use of progesterone in clinical settings associated with increased MMP-2 expression.

## Background

Matrix metalloproteinase-2 (MMP-2) plays a critical role in invasion, metastasis, angiogenesis and tissue remodelling [1]. The protein is widely expressed by a number of normal and transformed cells [2]. Recent studies have highlighted the important role of MMP-2 in the invasive potential of metastatic endometrial [3,4], ovarian [5] and trophoblast cells [6], as well as invasion of the normal trophoblast [7]. The ability of trophoblasts to infiltrate the uterine wall and to anchor the placenta to it, as well as their ability to infiltrate and adjust utero-placental vessels to pregnancy, is dependent upon MMP-2 secretion [8]. Abnormal MMP-2 secretion can also result in impaired implantation and is closely linked to several gestational pathologies, including repeated abortion [9], preeclampsia [10], gestational trophoblastic neoplasia and preterm labor [11,12]. The activity of MMP-2 is regulated at multiple levels, including gene transcription, translation, proenzyme activation and inhibition by the tissue inhibitors of metalloproteinases [13]. An extensive body of data has demonstrated a complex transcriptional regulatory network operative during both normal development and disease [[14-17], and [18]]. The human, and orthologous rat and murine MMP-2 proximal promoters lack a canonical TATA box and are composed of a relatively GC-rich region adjoining multiple transcriptional start sites. Functional binding sites for numerous transcription factors, including AP-1, AP-2, p53, nm-23β, and SP1/3 have been reported [14-19].

MMP-2 transcription is also regulated by reproductive hormones. Several studies have demonstrated induction of MMP-2 by estrogen in a variety of tissues, including vascular smooth muscle cells, glomerular mesangial cells and granulosa-lutein cells [20-22]. Estrogen responsiveness of human MMP-2 transcription was mapped to a half-palindromic binding site for the estrogen receptor (ERE), the activity of which was affected by an adjacent single nucleotide polymorphism [23]. In contrast, we, and others, have demonstrated that progesterone (PG) inhibits MM-2 expression in a variety of tissues [7,24-27]. This inhibitory effect of PG on MMP-2 expression occurs in the absence of a canonical progesterone response element (PRE) in the MMP-2 promoter. Progesterone has been part of the treatment protocol in number of conditions associated with enhanced MMP-2 secretion, including endometrial hyperplasia, imminent abortion and as luteal support in assisted reproduction [28-30]. Progesterone is also used as prophylactic treatment in women with a previous history of preterm labor [31].

Given the clinical importance of MMP-2 regulation by progesterone, we examined the transcriptional regulation of MMP-2 by progesterone using the model human JAR choriocarcinoma cell line. Our results support a novel mechanism by which progesterone inhibits MMP-2 expression. Within the context of human choriocarcinoma JAR cells basal transcription of MMP-2 is mediated by binding of primarily transcription factor SP4 to the MMP-2 proximal promoter. Progesterone suppresses MMP-2 transcription by reducing progesterone receptor (PR) and SP4 binding to the MMP-2 promoter. The inhibitory effect of progesterone on MMP-2 transcription may also result from enhanced proteasomal degradation of SP4/SP2.

## Methods

### Materials

Culture media and fetal calf serum (FCS) were obtained from Biological industries, Beit-Ha'Emek, Israel. Progesterone (P4) and Mifepristone (RU486) were obtained from Sigma (St. Louis, MO, USA); Antibodies were obtained from Santa Cruz Biotechnology (Santa Cruz, CA, USA). The BCA assay kit was obtained from Bio-Rad Laboratories, Inc, USA. All of the other reagents, unless otherwise specified, were purchased from Sigma.

### Cell culture

Human choriocarcinoma cell line (JAR) was obtained from the American Type Culture Collection (Manassas, VA). Cells were maintained in M199 medium containing 10% FCS in 60 mm culture plates, 24 wells and 96 wells kept in a humidified (37°C, 5% CO_2_) chamber. For the experiments described below, cells were trypsinized and harvested on reaching 80% to 90% confluence. The incubation period was 6–48 hours in the absence or presence of 0.1–10 μM progesterone (water soluble p-7556, Sigma, USA), and 10^-6 ^M Mifepristone (RU486, Sigma, USA) in serum free M-199 medium supplemented 1% penicillin/streptomycin and kept in 5% CO_2 _at 37°C.

### Gelatin zymography

To detect proteolytic activity in conditioned media collected after 48–72 h of culture, substrate gel electrophoresis (zymography) on gels containing gelatin as the substrate were used as described previously [7]. Identification of each gelatinase band was done in accordance to their molecular weight and commercial standards (gelatinize A and B, 7 μl; Oncogene Science; data not shown). These bands (pro-MMP) were quantified using the BioImaging gel documentation system (Dinco & Renum) endowed with TINA software (Raytest). The MMP secretion was expressed as a percentage of the control value.

### Western blot analysis

Western blot analysis was conducted as described previously [7]. To detect SP1, SP2, SP3, SP4, PR ubiquitin and GAPDH (for normalization), cell extracts (30 μg/lane) were diluted with 4× sample buffer (5% SDS and 20% glycerol in 0.4 M Tris, pH 6.8, containing 0.02% bromophenol blue) and subjected to 10% polyacrylamide gel electrophoresis. Transfer and blocking were performed as reported [7]. Blocked membranes were incubated for 1 h with either mouse anti-human SP1, SP2, SP3, SP4, ubiquitin and GAPDH antibody (1.0 μg/ml; Santa Cruz Biotechnology) or rabbit anti-human PR polyclonal antibody (1.0 μg/ml; sc-539; Santa Cruz Biotechnology) in 10% non-fat milk and Tris-buffered saline containing 0.01% Tween-20. The membranes were subsequently washed with Tris-buffered saline containing 0.5% Tween-20 and incubated for 1 h with horseradish peroxidase-conjugated anti-mouse rabbit secondary antibody (Jackson ImmunoResearch) in 10% non-fat milk and Tris-buffered saline containing 0.01% Tween-20, then detected by enhanced chemiluminescence (Amersham International) and quantified by densitometry as above.

### Protein assay

The total protein content of trophoblast cells was determined using a protein assay kit with BSA as the standard (Bio-Rad Laboratories, Inc. USA).

### Immunofluorescence

JAR cells were washed 3 times with PBS and fixed with 3.7% paraformaldehyde in PBS for 20 minutes at room temperature (RT) and then permeabilized for 3 minutes with 0.1% Triton X-100 in PBS. Incubation for 1 hour with blocking buffer (PBS supplemented with 3% FCS serum) and three washings in PBS. The cells were then incubated with primary antibodies (anti-SP4, sc-645 (3 μl into 700 μl PBS supplemented with 1.5% FCS), for 30 minutes at RT, followed by additional five washings in PBS. Cells were incubated in the dark for 30 minutes at RT with fluorescein-labelled phalloidin (AlexaFluor-488, A-12379, Molecular Probes) for F-actin (3 μl into 700 μl PBS supplemented with 1.5% FCS) and the secondary goat anti-rabbit IgG antibodies were conjugated with Alexa Fluor-546 (1 μl into 700 μl PBS supplemented with 1.5% FCS, A-11035, Molecular Probes) or secondary goat anti-mouse IgG antibodies were conjugated with AlexaFluor-633 (1 μl into 700 μl PBS supplemented with 1.5% FCS, A-21052, Molecular Probes). Following washing in PBS, stained cells were photographed using a confocal microscope. The photos were analyzed by Image Pro software that quantifies density per area.

### Semiquantitative RT-PCR

Reverse transcription-polymerase chain reaction was conducted with the following primers: MMP-2: forward, 5'-ACCTGGATGC-CGTCGTGGAC-3'; reverse, 5'-GTGGCAGCACCAGGGCAGC-3' (447-bp product). For normalization, we used the levels of the housekeeping gene GAPDH with the following primers: forward, 5'-TGATGACATCAAGAAGGTGGTGAAG-3'; reverse, 5'-TCCTTGGAGGCCATGTGGGCCAT-3' (240-bp product).

Total RNA was extracted from frozen samples with TRIzol reagent according to the manufacturer's instructions (Life Technologies, Inc.-BRL). The RNA concentrations were determined spectrophotometrically. A RT kit (Superscript preamplification system; Life Technologies, Inc.-BRL) was used in the synthesis and amplification of cDNA. Total RNA (5 μg) was denatured at 70°C for 10 min and then reverse transcribed in the presence of 25 ng/μl of oligo (deoxythymidine) primer (Life Technologies, Inc.), 2.5 mM MgCl2, 0.5 mM deoxy-NTPs, 10 mM dithiothreitol, and 10 U of ribonuclease H-reverse transcriptase (Superscript II RT; Life Technologies, Inc.) for 60 min at 42°C and 5 min at 95°C. Subsequently, 10 μl of the resulting cDNA were used as a template for PCR. The PCR was set up using 3 mM MgCl_2_, 50 pmol of each primer, and 2.5 U of Taq DNA polymerase (Sigma). The PCR conditions were 94°C for 2 min, followed by 35 cycles of 94°C for 30 sec, 60°C for 45 sec, and 72°C for 60 sec, with a 72°C extension for 10 min. After PCR, the products were resolved on a 2.5% agarose ethidium bromide gel. Images were captured with Polaroid film and quantified using BioImaging gel documentation system (Dinco & Renum) endowed with TINA software (Raytest).

### Plasmids

Firefly luciferase reporter plasmids incorporating either the human or rat MMP-2 promoters were prepared using the promoterless luciferase expression vector, pGL2-Basic (Promega). The human promoter pGL2-MMP-2 construct (denoted HpGL2-MMP2) extended to -1659 bp relative to the transcriptional start site was provided kindly by Dr. E.N. Benveniste [18]. The rat promoter pGL2-MMP-2 construct (denoted RpGL2-MMP-2) extended to -1686 bp relative to the translational start site. Serial deletion constructs of the RpGL2-MMP-2 extended to 1007, 573, 383, 321, 267 bp relative to the translational start site. A second series of serial deletion constructs extended from 1686 bp to 1433 bp, 1345 bp, 1262 bp and 1181 bp.

### Transient transfection and luciferase assays

Cells were transfected as described elsewhere in detail [7]. Briefly, 24 h before transfection, cells were plated in 24 or 96-well plates at a density of 6 × 10^5 ^cells per well. Cells were transfected by LipofectAMINE/Plus Reagent (Invitrogen) with full length or constructs plasmids (4 μg) or PGL2-basic (empty vector). The transfection was performed on JAR cells according to the manufacturer's directions (Invitrogen). Progesterone (1 μM) was added immediately after transfection. Luciferase assays of resultant cell lysates were performed 6 h after progesterone treatment according to the manufacturer's instructions (Promega Corp.). Luciferase reporter enzyme activity was determined by correcting for β-galactosidase and cell extract protein content as determined by the Bradford assay. Results from four independent experiments, each with duplicate wells, were averaged and presented as the mean ± SEM.

### Nuclear extract preparation

Nuclear and cytosolic extract proteins were prepared from the cell culture after the incubation period. Culture were lysed with ice-cold lysis buffer (10 mM Hepes [pH 7.9], 10 mM KCL, 1 mM EDTA, 1 mM dithiotheitol, 1 mM PMSF, 10 μg/ml of leupeptin, and 50 μg/ml of aprotinin). Suspensions were incubated for 15 min in 4°C, and Nonidet P-40 at a 0.4% final concentration was added. The cell suspension was centrifuged for 1 minute at 3000 rpm at 4°C, the supernatant containing the cytosolic fraction was removed; and the pellet was resuspended in the same lysis buffer, which contained 400 mM NaCl instead of KCl. After 15 min of incubation, the pellet suspensions were centrifuged for 5 min at 12000 rpm at 4°C. The nuclear extract was collected and stored at -20°C together with the cytosolic fraction until use.

### Electrophoretic mobility shift assay

**Figure 1:**
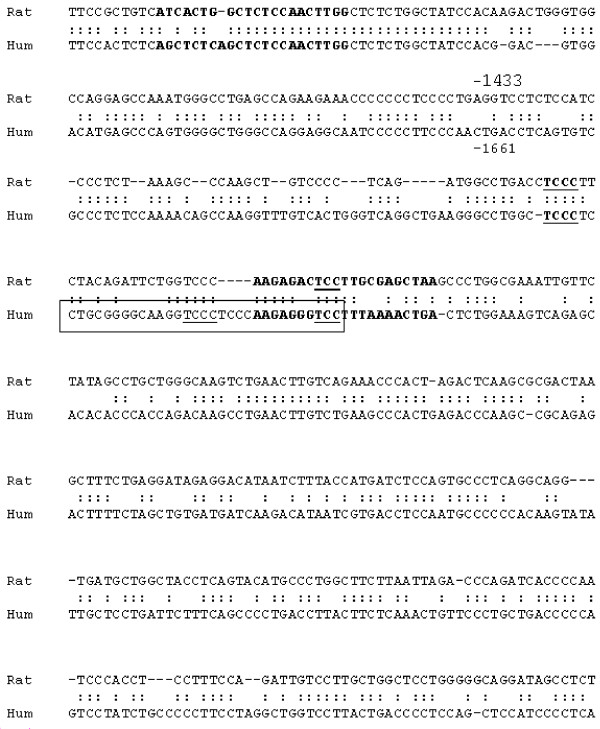
**Alignment of human and rat MMP-2 promoters**. Bold sequences represent primers used for chromatin immunoprecipitation. Boxed sequence details oligonucleotide sequence used for EMSA studies; underline sequences are the conserved SP1 consensus binding sites.

SP1 family of transcription factor activities were assessed by EMSA using double-stranded oligonucleotides corresponding to the SP1 consensus sequences (SP1, Santa Cruz Biotechnology, USA: sc-2501), or corresponding with the relevant SP1 binding site in the human promoter (5'-TCCTGCGGGGCAAGGTCCCTC-CAAGAGGGTCCTT-3') (Figure 1) Oligonucleotides were labelled with digoxigenin (Roche) using Terminal Transferase according to manufacturer's instructions in dig-labelling kit (Roche, Dyn Diagnostics Israel). Binding reactions were conducted by incubation of 6 μg of nuclear extract from JAR cells with digoxigenin-labelled oligonucleotide probes at 30°C for 30 min. in binding buffer containing 15 mM Hepes, 90 mM KCL, 6% glycerol, 3 mM DTT, 0.5 μg Poly [d (I-C)] and 0.4 ng oligonucleotide. To each sample 2.5 ml of loading buffer was added (60% TBE 0.25, 40% glycerol). Subsequently, DNA-protein complexes were separated from unbound oligonucleotides on a pre-electrophoresed 6% polyacrylamide gel and electrotransferred to positively charged nylon membranes (Roche, Dyn Diagnostics Israel). The DNA-protein complexes were fixed by baking 30 min at 120°C. Bands were detected with anti-digoxigenin-AP antibody and the chemiluminescent substrate CSPD (both Roche, Dyn Diagnostics Israel) on X-ray film. Sequence specificity of nuclear protein-oligonucleotide interaction was confirmed by competition with a mutated sequence (SP1, Santa Cruz Biotechnology, sc-2503) that was added to nuclear extracts for 10 minutes before addition of labelled probe.

### Chromatin immunoprecipitation (ChIP) Assay

Cells were fixed with 1% formaldehyde at 37°C for 10 min. Cells were washed twice with ice-cold PBS with protease inhibitors (1 mM phenylmethylsulphonyl fluoride, 1 μg/ml aprotinin, and 1 μg/ml pepstatin A), scraped and pelleted by centrifugation at 4°C. Cells were resuspended in a lysis buffer (1% SDS, 10 mM EDTA, and 50 mM Tris-HCl, pH 8.1), incubated for 10 min on ice, and sonicated to shear DNA. After sonication, lysate was centrifuged for 10 min at 13,000 rpm at 4°C. The supernatant was diluted in CHIP dilution buffer (0.01% SDS, 1% Triton X-100, 2 mM EDTA, 16.7 mM Tris-HCl, pH 8.1, 167 mM NaCl, and protease inhibitors). Primary antibodies were added and incubated overnight at 4°C with rotation. The immuno-complex was collected by protein A/G agarose beads and washed with low salt washing buffer (0.1% SDS, 1% Triton X-100, 2 mM EDTA, 200 mM Tris-HCl, pH 8.1, and 150 mM NaCl), high-salt buffer (0.1% SDS, 1% Triton X-100, 2 mM EDTA, 200 mM Tris-HCl, pH 8.1, and 500 mM NaCl), LiCl washing buffer (0.25 M LiCl, 1% NP40, 1% deoxycolate, 1 mM EDTA, and 10 mM Tris-HCl, pH 8.1), and finally 1' TE buffer (10 mM Tris-HCl, and 1 mM EDTA, pH 8.0). After that, the immuno-complex was eluted by the elution buffer (1% SDS, 0.1 M NaHCO3, and 200 mM NaCl) and the cross-links were reversed by heating at 65°C for 6 h. After reaction, the samples were adjusted to 10 mM EDTA, 20 mM Tris-HCl, pH 6.5, and 40 μg/ml proteinase K, and incubated at 45°C for 1 h. DNA was recovered and was subjected to PCR amplification of the rat MMP-2 promoter which contains the SP1 site at the -1433/-1342 bp region compliance with human promoter (Figure 1). The sequences for the primers are: forward 5-ATCACTGGCTCTCCAACTTGG-3,; reverse 5-TTAGCTCGCAAGGAGTCTCTT-3. The predicted size for the PCR product is 250 bp.

### Ubiquitinated SP4 protein immuno-precipitation

JAR cells were seeded into 60 mm tissue culture plates in maintenance medium and allowed to grow to approximately 90% confluence. Cells were treated with progesterone 1 μM for 2 h. Whole-cell extracts for the control and treatment group were obtained using RIPA buffer (50 mM Tris-HCL, 1% Nonidet P-40, 0.25% sodium deoxycholate, 150 mM NaCl, and 1 mM EDTA) with the addition of protease inhibitor cocktail. Duplicate aliquots of 500 μg were used for the experiments. Cell extracts were diluted in ice-cold PBS containing protease inhibitor cocktail to a final volume of 1 ml, followed by the addition of 30 μl of protein A/G PLUS-agarose beads (Santa Cruz, USA). The reactions were placed on a rocker at 4°C for 3 h, followed by 600 g centrifugation at 4°C for 5 min. A 900-μl aliquot of supernatant from each sample was transferred into a new Eppendurf tube on ice. Rabbit polyclonal anti-Sp4 (1 μg), SP1 (1 μg), SP2 (1 μg), SP3 (1 μg), or normal rabbit IgG (1 μg) was added to either control or treatment set, followed by the addition of 30 μl of protein A/G PLUS-agarose beads. The samples were then placed on a rocker at 4°C overnight, followed by centrifugation at 2500 rpm at 4°C for 5 min. The supernatant was removed by aspiration and the immuno-precipitates were washed with two cycles of 1 ml of ice-cold RIPA buffer followed by 1 ml of ice-cold PBS using centrifugation at 600 g at 4°C for 5 min. The agarose pellet was resuspended in 40 μl of loading buffer, boiled, and centrifuged. The supernatant was separated by SDS-10% PAGE, electrophoresed. Total protein (input) was tested for either, SP1, SP2, SP3, SP4, PR, ubiquitin and GAPDH. The immuno-precipitant was tested for the presence of ubiquitin.

### Statistical analysis

Results are expressed as the mean ± SEM of 3 to 4 independent experiments where each treatment was performed in duplicate. Statistical analysis was performed using the SPSS statistical software, guided by the statistician annalist from our central statistical centre. A level of P < 0.05 was considered to be significant.

## Results

### Progesterone inhibits MMP-2 expression by JAR choriocarcinoma cells

**Figure 2:**
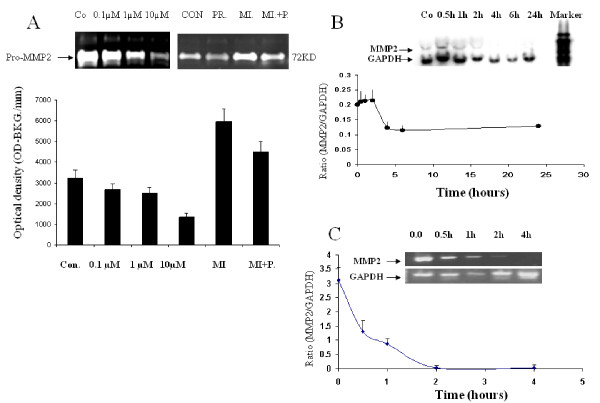
**Progesterone suppresses JAR MMP-2 secretion and synthesis**. (A) Secretion of pro-MMP-2 by JAR cells after 48 hours incubation in the absence or presence of progesterone alone (1 μM–10 μM) or in combination with Mifepristone (MI; 10^-6 ^M). Results represent mean ± SEM from 4 independent experiments *P < 0.05 versus untreated cells. Total RNA of MMP-2 isolated form JAR cells treated with progesterone (1 μM) for the indicated times was assessed by Northern blot (B) and semiquantitative reverse transcription-PCR (C). The data are presented as the ratio of MMP-2 to GAPDH. The values represent the mean ± SEM from 4 different experiments performed in duplicates.

The effect of progesterone on MMP-2 expression in JAR human choriocarcinoma cell line was conducted. JAR cells (2 × 10^5^) were incubated 48 h in the absence or presence of progesterone (0.1–10 μM), and media were analyzed by zymography for gelatinase secretion. Progesterone significantly decreased secretion of pro-MMP-2 in a dose-dependent manner (P < 0.05). Incubation with progesterone (1,10 μM) decreased pro-MMP-2 secretion by 22 percent (P < 0.01) and by 58.7 percent (P < 0.01) respectively (control 3245 ± 360, 1 μM 2530 ± 246 10 μM 1340 ± 201). Incubation of progesterone with 10^-6 ^M Mifepristone, a progesterone antagonist, abolished the inhibitory effect of progesterone on pro-MMP-2 (Figure 2A).

The time course of progesterone-dependent regulation of MMP-2 gene expression was examined. Transcript expression of MMP-2 in JAR choriocarcinoma trophoblast cells treated with progesterone for several time intervals is presented using Northern blot (Figure 2B) and semi-quantitative RT-PCR (Figure 2C). Progesterone (1 μM) significantly decreased MMP-2 transcript expression level in a time-response manner (P < 0.05). To compare MMP2 mRNA relative expression levels between groups, we analyzed the ratio of each independent experiment between the expression level of either MMP2 and the house keeping gene GAPDH from the same tissue under the same treatment. Inhibition was observed after 4 h of incubation.

### Progesterone inhibits MMP-2 via transcriptional repression

**Figure 3:**
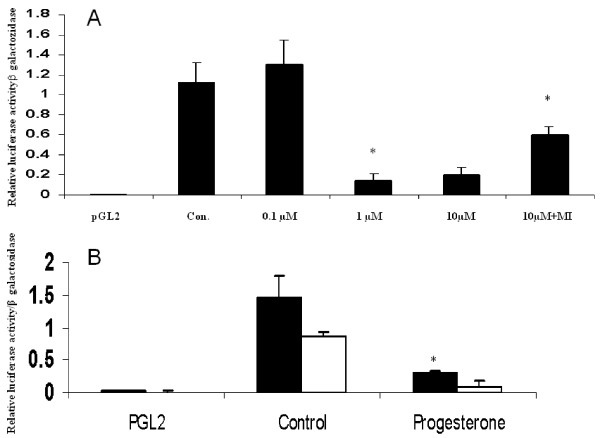
**MMP-2 luciferase reporter activities: effects of progesterone**. (A) JAR cells transfected with control pGL2 vector or pGL2 vector containing the human MMP-2 promoter in the presence or absence of progesterone (1 μM–10 μM) or in combination with RU486 (MI, 10–6 M). (B) JAR cells transfected with pGL2 containing the full length (1686 bp) human or rat promoters in the absence or presence of progesterone (1 μM). Results represent mean ± SEM from four independent experiments; * P < 0.05. Black bars: human MMP-2 promoter; white bars: rat MMP-2 promoter.

We next performed MMP-2 promoter activity assays to determine if the suppression of MMP-2 synthesis by progesterone occurs at the transcriptional level. Maximal inhibition of human MMP-2 promoter activity was observed with 1 μM progesterone (Figure 3A). Following incubation of progesterone and 10^-6 ^M Mifepristone inhibitory effect of progesterone was abolished (Figure 3A). Similar and significant decreases in both rat and human MMP-2 promoter activities were observed following the addition of 1 μM progesterone (80.1% ± 0.3 and 81.3% ± 0.23, respectively, P < 0.01, Figure 3B). Thus, progesterone inhibits MMP-2 synthesis by direct transcriptional repression.

### Progesterone suppresses MMP-2 promoter activity via SP1 site

**Figure 4:**
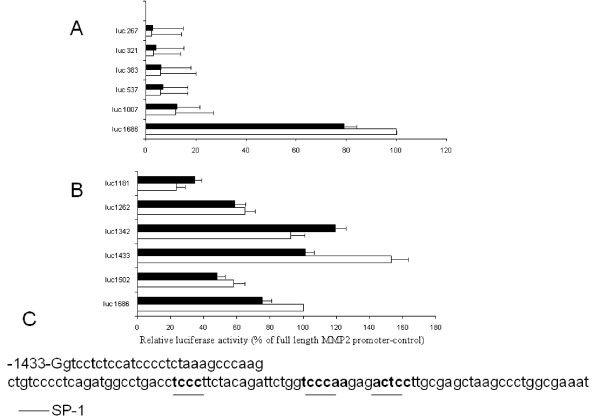
**Serial deletion mapping of region in rat MMP-2 promoter responsible for progesterone inhibition of MMP-2 transcription**. (A) Coarse promoter deletion mapping; (B) Fine promoter deletion mapping. (C) Sequence from -1433 to 1342 bp containing progesterone-response region and three potential SP1 binding sites (underlined). White bars: untreated cells (control); black bars: treated cells with progesterone (1 μM).

Potential regulatory regions in the rat MMP-2 promoter responsible for the inhibition by progesterone were analyzed in a series of 5-deletion constructs. A full length rat MMP-2 luciferase reporter construct (-1686 bp), or defined deletions constructs, were transfected into JAR cells treated in the absence or the presence of progesterone (1 μM). As shown in Figure 4A, addition of progesterone significantly decreased luciferase reporter activity in the region between -1007 bp and -1686 bp relative to the MMP-2 translational start site.

In MMP-2 5'-deletion construct extending to -1502 bp the inhibitory effect of progesterone was slightly reduced, however in the -1433 bp deletion construct progesterone significantly increased its ability to inhibit luciferase activity (153 ± 10.1 versus 101.2 ± 7.1 percent; P < 0.05), (Figure 4B). This inhibitory effect of progesterone was abolished in the -1342 bp deletion construct (92.8 ± 12 and 119.3 ± 11 percent of luciferase activity; P < 0.05). The progesterone-responsive element should be therefore located between -1432 to -1342 bp region of the rat-MMP-2 promoter. In the -1262 bp and -1181 bp deletion constructs no effect of progesterone was observed.

Transcription factor binding site analysis [32] localized three potential SP1 binding sites in this sequence that is conserved in the homologous human MMP-2 promoter sequence (Figure 1). The sequence analyses suggest that the inhibitor effect of progesterone on MMP-2 transcription may involve interaction of members of the SP1 transcription factor family with specific binding sites in the 91 bp sequence extending between -1433 and -1342 bp of the rat MMP-2 promoter and the corresponding region of the human MMP-2 promoter.

**Figure 5:**
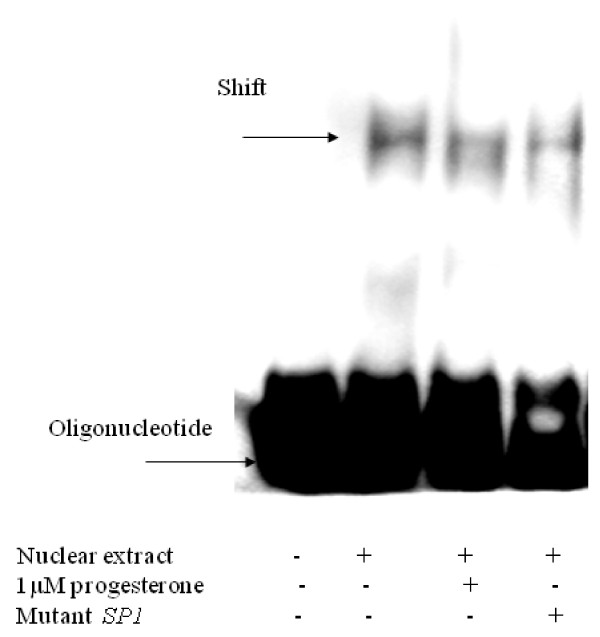
**EMSA of JAR cell nuclear extracts with DNA-nuclear protein complex binding to sequence relevant SP1 binding site in the human promoter: effect of progesterone**. Nuclear proteins were isolated from JAR cells cultured with or without progesterone (1 μM). The specificity of the DNA-nuclear protein complex was tested by competition with mutant oligonucleotide. The figure represents five separate experiments. The positions of free oligonucleotide and shifted oligonucleotide are indicated by arrows.

EMSA was performed to explore the interaction of SP1 family of transcription factors with these sites. As demonstrated in Figure 5, the nuclear proteins extracted from JAR cells formed DNA-protein complex with the DNA probe corresponding to the *SP1 *oligonucleotide corresponding with the relevant sequence in the human promoter (Figure 5). DNA-protein complex was decreased in the presence of progesterone (1 μM). The addition of mutant DNA probes effectively reduced the signal intensity of the DNA-protein complex. Similar was obtained with *SP1 *oligonucleotide corresponding with the consensus binding site of SP1 (data not shown).

### Progesterone reduces transcription factors binding to the response element on the MMP-2 promoter

**Figure 6:**
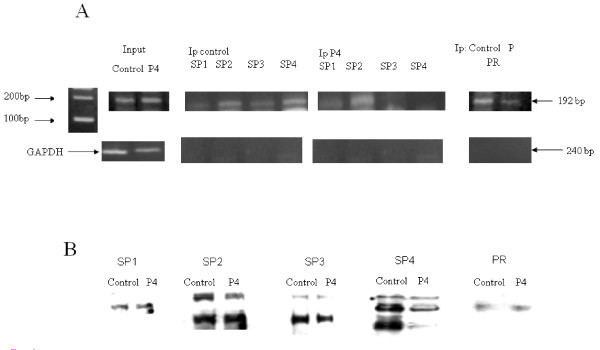
**Chromatin immunoprecipitation analysis of SP1-4 and PR interactions with the progesterone regulatory region within a genomic context**. Progesterone reduces JAR nuclear SP4 concentration. (A) Chromatin immunoprecipitation was performed as detailed in Experimental Procedures using sheared chromatin prepared from JAR cells after four hours incubation in the presence or absence of progesterone (1 μM). Treatment with progesterone results in decreased binding of SP4 and PR to the progesterone regulatory region of the MMP-2 promoter. (B) Western blots of JAR cell nuclear extracts from cells after four hours incubation in the presence or absence of progesterone (1 μM). Progesterone treatment specifically reduces JAR cell nuclear SP4 levels.

The effect of progesterone (1 μM) was studied by ChIP assay of the classical SP1 family of transcription factors and the PR. Cells were fixed by formaldehyde, and soluble chromatin was purified by sonication of cross-linked nuclei. Chromatin fragments in the range of 500–1000 bp were immunoprecipitated by specific antibodies (SP1, SP2, SP3, SP4 and PR). Purified genomic DNA from immunoprecipitated chromatin was subjected to semi-quantitative PCR using primers covering the 250-bp -1433/-1342 region of the rat-*MMP-2 *promoter corresponded with human promoter (Figure 1). Immunoprecipitation by IgG was used as a negative control. Input of both the control and progesterone treated cells revealed similar amounts of GAPDH (as internal control, Figure 6A). In the control cells, SP3 and SP1 bands were minimally detectable, while SP2 and SP4 bands were readily evident. PR was also found to bind to the same region in the MMP-2 promoter. Thus, in the basal state in an intrinsic genomic context, the MMP-2 promoter is occupied by primarily SP2/PR and SP4/PR complexes. In the presence of progesterone there was a reduction in SP4 and in PR binding to the DNA. No significant change was observed in SP1, SP2 and SP3 following progesterone treatment. Therefore, progesterone reduces basal MMP-2 transcription primarily through a reduction of SP4/PR complex binding to the intrinsic MMP-2 promoter.

In order to study the possible mechanism for the reduction in SP4 binding following progesterone treatment, western blot for the classical SP1 family was performed with and without progesterone. Results (Figure 6B) show that SP1, SP2, SP3 and SP4 are expressed in JAR cells. Following treatment with progesterone only SP4 expression is reduced significantly. These results suggested that progesterone not only affects SP4/PR binding to the intrinsic MMP-2 promoter but may also affect SP4 protein levels, per se.

### Role of progesterone on SP4 degradation

**Figure 7:**
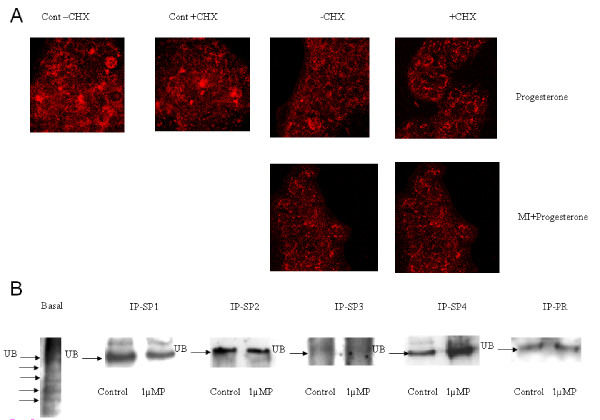
**Progesterone reduces SP4 through enhanced degradation and ubiquitin binding rather that inhibition of protein synthesis**. (A) Immunofluorescence staining of JAR cells for SP4 protein in the presence or absence of progesterone and cycloheximide (CHX). Red staining represents SP4 protein. (B) Western blot analysis for Ubiquitin binding to the corresponding SP protein of immunoprecipated SP1-4 and PR proteins from cells cultured for four hours in the presence or absence of progesterone (1 μM). Treatment with progesterone specifically promotes Ubiquitin binding to the SP4 protein.

To test the possibility that the reduction of SP4 protein reflects enhancement in its degradation rate rather then, synthesis inhibition, cells were exposed to cycloheximide (CHX; 10 μg/ml), a syntactic protein inhibitor, in the presence or absence of progesterone. The addition for 1 hour of progesterone alone or progesterone with CHX reduced SP4 expression (Figure 7A) indicating that the reduction in SP4 expression is most probably due to SP4 degradation. The addition of RU486 did not change the progesterone effect on SP4 expression, suggesting a non-classical role for progesterone activity on SP4 degradation.

The effects of progesterone on Ubiquitin binding capacity to the SP1, SP2, SP3, SP4 and PR proteins were investigated. (Figure 7B). Whole-cell lysates from control and progesterone-treated groups were initially immunoprecipitated with a control IgG or antibodies to SP1, SP2, SP3, SP4 and PR. Immunoprecipitated fractions were separated by SDS-polyacrylamide gel electrophoresis and immunoblotted with anti-ubiquitin antibodies. Antibodies to GAPDH were used as an internal control (data not shown). The addition of progesterone induced specific ubiquitin binding only to the SP4 protein.

## Discussion

Synthetic progestational agents have been used extensively in invasive gynecological pathologies such as endometriosis [33] and endometrial hyperplasia [28]. Progesterone is a paradoxical hormone having either stimulatory or inhibitory effects, depending on the tissue in question and the dose and treatment regimen [34,35]. Classical P4 activity is mediated by interaction of the hormone with the two classical intracellular progesterone receptor (PR) isoforms PR-A and PR-B [34,35]. Both isoforms, encoded by a single gene independently regulated by separate promoter, regulate different subsets of genes. PR frequently acts as ligand-inducible transcription factor in the respective target cell by binding to specific progesterone response elements (PRE) in the promoter of target genes [36]. The mechanisms of progesterone are only partial explained by their isoforms profile ratio expression.

Recent reports indicate that progesterone can also regulate promoters lacking a canonical progesterone response element [37-40]. In these cases, progesterone receptors interact indirectly with the promoter through complex formation with various transcription factors, including several members of the SP1 transcription factor family [38,41,37].

We have previously reported that progesterone inhibits MMP-2 synthesis in reproductive tissues [7]. JAR choriocarcinoma cells synthesize high levels of MMP-2, which is responsible for the highly invasive nature of these cells. In this report we demonstrate that this inhibitory effect of progesterone occurs at a transcriptional level in the absence of a canonical PRE in the human or rat MMP-2 promoters and this inhibitory effect is reversed by Mifepristone. Using a combination of techniques, we mapped the region responsible for high level MMP-2 transcription in JAR cells to a GC-rich region in the MMP-2 proximal promoter which contained several potential SP1 transcription family binding sites. In the basal state the MMP-2 promoter is occupied by SP2/PR and SP4/PR complexes. Addition of progesterone reduces MMP-2 transcription through a reduction in SP4 and in PR binding to the intrinsic MMP-2 promoter.

In addition to displacement of SP4/PR binding from the MMP-2 promoter, progesterone also affects the net concentration of the SP4 protein. Addition of progesterone to JAR cells specifically promoted degradation of the SP4 protein, while there was no effect on SP1-3 protein degradation. The failure of Mifepristone (progesterone receptor antagonist) to reverse this progesterone effect, together with lack of effect by CHX, might suggest that progesterone is involved, via non classical mechanism, in SP4 protein degradation rather than inhibition of the protein synthesis. The ability of progesterone to enhance specific ubiquitin binding to SP4 protein after four hours incubation might suggest that ubiquitination of the SP4 protein would result in enhanced rates of SP4 protein degradation in the proteasome, thereby reducing SP4 protein levels and the ability to form transactivating SP4/PR complexes. Repression of SP4-mediated transactivation via enhanced proteasomal degradation may be a more common event than previously recognized. Adelrahim and Safe, [42], recently demonstrated that cyclooxygenase inhibitors decrease vascular endothelial cell growth factor expression via enhanced degradation.

## Conclusion

Progesterone reduces PR and SP4 binding to the MMP-2 promoter and promotes SP4 degradation, thereby suppressing MMP-2 promoter transcription. These findings provides further example for the complex nature of progesterone-dependent transcriptional regulation, particularly in the absence of a canonical PRE in the proximal promoter. To our knowledge, the mechanisms of progesterone-mediated MMP-2 transcriptional suppression are unique and provide a firm biological basis for the clinical application of progesterone in the treatment of gynecologic and obstetric disorders associated with increased MMP-2 expression.

## Competing interests

The authors declare that they have no competing interests.

## Authors' contributions

SG carried out the laboratory work, participated in conceiving and designing of the study. Performed the statistical analysis and drafted the manuscript. DHL took part in the design, provided the rat promoter deletions and helped in drafting of the manuscript. ES Conceived and design the study analysed the results and edited the manuscript. All authors read and approved the final manuscript.
